# Haemoproteosis lethality in a woodpecker, with molecular and morphological characterization of *Haemoproteus velans* (Haemosporida, Haemoproteidae)

**DOI:** 10.1016/j.ijppaw.2019.07.007

**Published:** 2019-07-19

**Authors:** Tierra C. Groff, Teresa J. Lorenz, Rocio Crespo, Tatjana Iezhova, Gediminas Valkiūnas, Ravinder N.M. Sehgal

**Affiliations:** a San Francisco State University, Biology Department, 1600 Holloway Ave, San Francisco, CA, 94312, USA; b U.S. Department of Agriculture, Forest Service, Pacific Northwest Research Station, 3625 93rd Ave SW, Olympia, WA, 98512, USA; c North Carolina State, College of Veterinary Medicine, 1060 William Moore Dr, Raleigh, NC, 27607, USA; d Institute of Ecology, Nature Research Centre, Akademijos Str. 2, LT-08412, Vilnius, Lithuania

**Keywords:** *Haemoproteus velans*, Molecular and morphological characterization, Mortality, Picidae, White-headed woodpecker, Radio-tagging

## Abstract

A juvenile White-headed woodpecker (*Dryobates albolarvatus*) fitted with a radio tag was located dead at approximately 22-days post-fledging in Yakima county in central Washington in July 2015. Postmortem examination revealed an enlarged liver and spleen plus evidence of iron sequestration. Microscopic examination observed young gametocytes within the cytoplasm of erythrocytes, and exo-erythrocytic meronts within the cytoplasm of capillary endothelial cells, hepatocytes, and myocytes, and free in the tissues. These attributes implicated a haemosporidian infection that likely resulted in mortality. Subsequent sampling results of local woodpecker species in the same area during the breeding season in June–July 2016 and May–July 2017 showed other individuals infected with *Haemoproteus* parasites. Nested Polymerase Chain Reaction (PCR), sequencing, and microscopic analyses for avian haemosporidians revealed infections with *Haemoproteus velans* (Haemosporida, Haemoproteidae). This parasite was characterized molecularly and morphologically. This is the first report of a haemosporidian infection in a White-headed woodpecker anywhere in its range, and the first reported suspected mortality from haemoproteosis for a woodpecker (Piciformes, Picidae). The use of radio-tagged birds is an asset in wildlife haemosporidian studies because the effect of the pathogen can be monitored in real time. Additionally, this methodology provides opportunities to collect fresh material for microscopic and histological examination from wild birds that have died from natural causes.

## Highlights

• Radio-tagged juvenile White-headed woodpecker found shortly after death.
• Necropsy revealed likely died from complications of an avian haemosporidian, *Haemoproteus velans*, infection.
• *Haemoproteus* genus not thought to cause mortality in wild birds.
• First molecular characterization of *H. velans* and new morphological characterization.
• Avian haemosporidian infections increase in severity as climate change affects imperiled species.

## Graphical abstract

**Figure:**
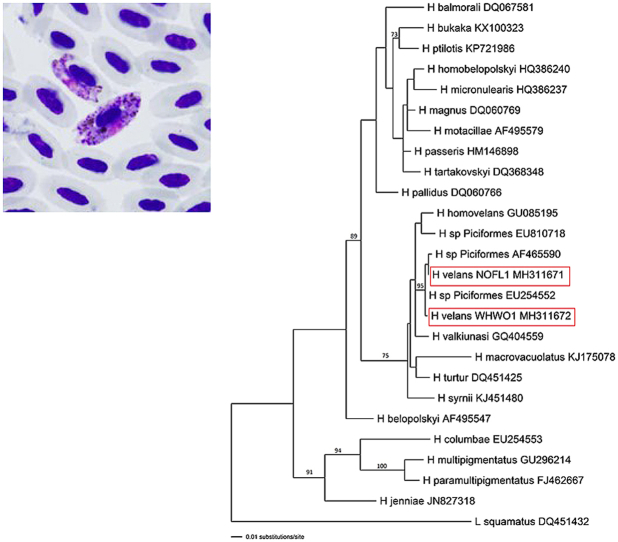

## 1 Introduction

Prevalent worldwide, avian haemosporidian parasites (Apicomplexa, Haemosporida) belonging to the genera *Leucocytozoon, Plasmodium, Haemoproteus*, and *Fallisia* infect a majority of terrestrial avian species (Valkiūnas, 2005). Though morbidity can be high in some populations, mortality is difficult to detect because sick individuals are secretive and/or are rapidly eliminated by predators (Lachish et al., 2011). Current sampling techniques favor capturing wild individuals with light (chronic) parasitemia (Valkiūnas, 2005; Mukhin et al., 2016). These birds have already survived the acute stage of infection and are healthy enough to be mobile in the environment, breed, and migrate (Bennett et al., 1993b; Mukhin et al., 2016). Locating wild haemosporidian caused fatalities presents logistical challenges, leading to biased detection of mortality rates (Holmes, 1982; Bennett et al., 1993b; Valkiūnas, 2005). These sampling biases lead to the assumption that avian haemosporidian infections are relatively benign in wild populations (Bennett et al., 1993b).

Haemosporidian infections of *Leucocytozoon* and *Plasmodium* are common and can cause high mortality in domesticated birds in the families Anseriformes, Columbiformes, Galliformes, and Struthioniformes (Bennett et al., 1993a; Valkiūnas, 2005), wild birds kept in captivity (Bennett et al., 1993b; Valkiūnas, 2005; Bueno et al., 2010; Vanstreels et al., 2015), and novel introductions (Warner, 1968; Van Riper et al., 1986; Bennett et al., 1993b; Argilla et al., 2013; Marzal et al., 2015). *Haemoproteus* infections are also common but are generally not thought to cause mortality in adapted species (Bennett et al., 1993b). However, there have been an increasing number of cases of severe disease and mortality in experimentally infected domesticated birds (Atkinson et al., 1986, 1988; Cardona et al., 2002; Valkiūnas and Iezhova, 2017), naturally infected birds in captivity (Earle et al., 1993; Ferrell et al., 2007; Donovan et al., 2008; Olias et al., 2009; Cannell et al., 2013) and case studies with a few sick individuals (Atkinson and Forrester, 1987; Peirce et al., 2004). *Haemoproteus* megaloschizonts in skeletal muscle produce severe, acute hemorrhagic myositis which is associated with severe disease and death (Atkinson et al., 1988; Cardona et al., 2002; Olias et al., 2009; Valkiūnas and Iezhova, 2017). The majority of documented *Haemoproteus* fatalities in wild populations have been represented by single individuals, so it is difficult to determine what factors contribute to lethality.

There are over 150 species in the genus *Haemoproteus* documented to infect a range of avian hosts (Valkiūnas, 2005; Dimitrov et al., 2014). All haemosporidians use dipteran insect vectors as the definitive host, with haemoproteids of the subgenus *Parahaemoproteus* utilizing biting midges (Diptera: Ceratopogonidae) (Atkinson et al., 2008). Two species of *Haemoproteus* have been reported in North American woodpeckers, *H. borgesi* (Pung et al., 2000) and *H. velans* (Coatney and Roudabush, 1937; Khan and Fallis, 1971; Greiner et al., 1975, 1977; Pung et al., 2000; Schrader et al., 2003; Valkiūnas, 2005). *H. borgesi* can be readily distinguished from *H. velans* primarily due to the shape of its fully-grown gametocytes, which are broadly halteridian in the former species, but are predominantly markedly circumnuclear in the latter parasite (Greiner et al., 1977; Valkiūnas, 2005). *H. borgesi* remains non-characterized molecularly a detailed re-description of its blood stages is nedded. There are no records of mortality caused by any *Haemoproteus* species or parasites of other genera of haemosporidians in woodpeckers despite the reports of 11 species harboring infections (Table 1). Most of the unidentified historical *Haemoproteus* samples are thought to be *H. velans* (Greiner et al., 1977).

**Table 1:** Documented North American haemosporidian infections in Piciformes (1937–2018)^a^.

Species	No. examined	No. infected	*Leucocytozoon*	Infected with *Haemoproteus*	*Plasmodium*
*Colaptes auratus*	198	108	22	78	8
*Picoides arcticus*	9	4	1	3	0
*Dryobates borealis*	70	1	0	1	0
*D. pubescens*	140	14	0	14	0
*D. villosus*	44	26	3	23	0
*Hylatomus pileatus*	1	1	0	1	0
*Melanerpes erythrocephalus*	19	2	0	2	0
*M. formicivorus*	19	0	0	0	0
*M. carolinus*	243	64	0	63	1
*Sphyrapicus nuchalis*	10	1	0	1	0
*S. varius*	39	13	1	11	1
Total	792	234	27	197	10
Overall prevalence (%)		29.5	3.4	24.9	1.2

a Coatney and Roudabush (1937); Coatney (1938); Huff (1939); Herman (1944); Bennett and Fallis (1960); Collins et al., (1966); Khan and Fallis (1971); Greiner et al., 1975, Greiner et al., 1977; Pung et al., 2000; Ricklefs and Fallon (2002); Schrader et al., (2003); Martinsen et al., (2008); Astudillo et al., (2013); Medeiros et al., (2014), 2015; Ellis et al., (2015); Walther et al., (2016); Smith et al., (2018).

*Haemoproteus velans* was first described by Coatney and Roudabush (1937) in Northern Flickers (*Colaptes auratus)*. This parasite is readily distinguishable by the morphology of its fully-grown gametocytes, which are circumnuclear and slightly displace laterally the nucleus of infected erythrocytes (Greiner et al., 1977; Valkiūnas, 2005). The gametocytes often possess numerous prominent volutin granules. Additionally, when fully grown, the gametocytes completely encircle the nucleus and occupy the available cytoplasm. Morphometric characterization of gametocytes of this parasite remains incomplete (Coatney and Roudabush, 1937; Greiner et al., 1977; Valkiūnas, 2005). Two biting midge species, *Culicoides stilobezzoides* and *Culicoides sphagnumensis* have been identified as vectors in experimental conditions (Khan and Fallis, 1971).

Woodpeckers are part of an important keystone guild of primary cavity excavators (Raphael and White, 1984; Drever et al., 2008). Nest and roost cavities excavated by woodpeckers provide nests and shelter sites for many small-bodied secondary cavity users (Blendinger, 1999; Aitken and Martin, 2007; Tarbill et al., 2015). Woodpeckers have been shown to help control forest insect populations (Fayt et al., 2005) and may help disperse fungi that act as agents of decay (Jusino et al., 2016). Documented woodpecker mortalities attributed to pathogens have been represented by case studies describing single individuals (Gerhold and Yabsley, 2007; Siegel et al., 2012; Garigliany et al., 2014; Jokelainen and Vikøren, 2014), leading to little baseline information on etiological agents of mortality. Health factors, such as haemosporidian infections, impacting population growth rates or the ability of the species to maintain healthy population levels could have a significant impact on forest ecosystem function. The White-headed woodpecker (*Dryobates albolarvatus*) is believed to be declining in many areas due to habitat loss, particularly in the northern parts of its range (Garret et al., 1996). Listed as an endangered species in Canada and considered a species of special concern in some areas (COSEWIC, 2010; WDFW, 2013; ODFW, 2016), White-headed woodpeckers have not been sampled in past studies of haemosporidians, due to their rarity and restricted geographic range (Altman, 2000). To conserve populations of these important keystone species, more information about mortality factors could be useful for managers. Here, we identify *H. velans* as the cause of death in a juvenile White-headed woodpecker and as the source of chronic infections in adult Northern Flickers from Eastern Washington, suggesting that avian haemoproteosis caused organ pathologies may be more important in the population dynamics for woodpeckers than previously envisioned.

## 2 Materials and methods

### 2.1 Radio-telemetry field methods

During a study on post-fledging dispersal, 54 nestling White-headed woodpeckers were captured and radio-tagged in Yakima County, Washington (approximately 46° 45′ N, 120° 58′ W) from 2014 to 2017. Individuals were tracked every 1–4 days while they were still dependent on their parents, which was defined as starting on the day nestlings fledged and ending three days after the bird was last seen begging for food from a parent. Point locations were recorded when birds moved. We calculated home ranges for the dependence period using the minimum convex polygon method (White and Garrot, 1990; Millspaugh and Marzluff, 2001).

### 2.2 Blood sampling field methods

We sampled live woodpeckers during the radio-telemetry study in June–July 2016 and May–July 2017 during the breeding season. We searched known territories of White-headed woodpeckers and Black-backed woodpeckers (*Picoides arcticus*) for nests throughout the nesting season. Northern Flickers and Hairy woodpeckers (*Dryobates villosus*) nests located during these searches were included to increase our sample size. Adults were captured using targeted mist-nets positioned in front of the nest cavity. We set up nets just after a parent had fed the nestlings to limit the amount of time they were kept away from foraging. Nestlings were sampled from the nest when they were an estimated 1–4 days from fledging using the hole saw method described by Ibarzabal and Tremblay (2006). All birds were removed from the net or nest cavity and were weighed, measured, and banded in compliance with the Ornithological Council Guidelines for the Use of Wild Birds in Research (Fair et al., 2010) and U.S. Department of Agriculture, Forest Service, Institutional Animal Care and Use Committee (Proposal number 2016–007). Adult birds were not aged, and nestlings were between 18 and 25 days old.

We obtained 25–50 μl of blood from each bird via brachial venipuncture with a sterile 27-gauge needle. Blood was then stored in lysis buffer (10 mM Tris-HCL pH 8.0, 100 mM EDTA, 2% SDS) at ambient temperature while in the field and preserved at −20 °C in the laboratory until further processing (Sehgal et al., 2001). Two to three blood smears from each individual were prepared in the field using established techniques (Bennett, 1970; Valkiūnas, 2005), fixed in methanol, and stained with Giemsa and examined microscopically following established protocols in the laboratory (Valkiūnas et al., 2008b). Voucher preparations of *H. velans* from its vertebrate type host, the Northern Flicker, were deposited in Nature Research Centre, Vilnius, Lithuania (accession nos. 49035 and 49036 NS).

### 2.3 Post-mortem white-headed woodpecker examination

The Avian Health and Food Safety Laboratory at Washington State University in Payallup, Washington performed a postmortem examination on the dead juvenile White-headed woodpecker. Collected samples of multiple tissues were fixed in 10% neutral buffered formalin for 48 h. All tissues were dehydrated in graded alcohols, cleared in xylene, embedded in paraffin, sectioned at 4 μm, stained with hematoxylin and eosin. The sections were examined under a light microscope from 2x to 100x magnification. In addition, formalin-fixed skeletal muscle, kidney, spleen, liver tissues were processed for transmission electron microscopy (TEM) following (Widáhn and Kindblom (1988). Briefly, selected areas of these tissues were cut from the paraffin block, deparaffinized in xylene and processed into Eponate 12s epoxy resin (Ted Pella Inc., Redding, CA). Thick sections were cut, mounted on glass slides, and stained with Toluidine Blue O and examined by light microscopy. Thin sections were cut, mounted on 150-mesh copper grids, stained with 6% uranyl acetate in 75% ethanol, poststained in Reynold lead citrate, and examined with a transmission electron microscope. Initial PCR testing conducted by the Avian Health and Food Safety Laboratory for a haemosporidian infection was inconclusive. Since macroscopic findings were similar to those found in other *Haemoproteus* infection fatalities, the case was referred San Francisco State University for further molecular testing.

### 2.4 Parasite molecular analysis

Molecular analysis took place at San Francisco State University. DNA extraction from muscle tissue from the deceased White-headed woodpecker and blood samples from sampled live birds used the commercial DNA extraction kit *Wizard SV Genomic DNA Purification System* (Promega Corporation, Madison, WI) and followed the manufacturer's protocol. We confirmed successful DNA extraction using primers that amplified the brain-derived neurotrophic factor (Sehgal and Lovette, 2003). Nested PCR screened for a partial sequence of the mitochondrial cytochrome *b* (cyt *b*) gene from *Plasmodium* and *Haemoproteus* using the primers HaemNF/HaemNR2-HaemF/HaemR2 (Bensch et al., 2000; Waldenström et al., 2004) and from *Leucocytozoon* using primers NF/NR3-FL/R2L (Hellgren et al., 2004). All reactions were carried out in 25 μl reactions and accompanied by negative (ddH2O) and positive controls (samples from infected birds previously confirmed by sequencing and microscopy). We visualized the resulting PCR product on a 1.8% agarose gel to check for positive infection. Positive PCR products were sequenced by Elim Biopharmaceuticals Inc. (Hayward, CA).

### 2.5 Phylogenetic analysis

Geneious software was used (v.11.0.4) (https://www.geneious.com, Kearse et al., 2012) to edit and align sequences. After trimming primers, a sequence length of 480 base pairs was obtained. We classified a unique sequence as differing from other sequences by one or more nucleotides (Martinsen et al., 2006; Hellgren et al., 2007). By comparing samples to other haemosporidian sequences available using the National Centre for Biotechnology Information's Basic Local Alignment Search Tool (NCBI Resource Coordinators, 2016) and the MalAvi Database (Bensch et al., 2009), two distinct *Haemoproteus* lineages were identified and were deposited in GenBank (MH311671, MH311672). We generated a Maximum-likelihood (ML) tree in PAUP* (v.4.0a.b161) (Swofford, 2018) incorporating sequences from this study and 18 reference sequences submitted to GenBank. The analyses only included reference sequences that have well-established morphological species identifications, which were based on microscopic examination of blood films. A sequence from a Downy woodpecker (*Dryobates pubsescens*) (EU254552) with a suspect morphological identification of *H. picae* (Dimitrov et al., 2014) was included due its genetic similarity to our lineages. Additionally, we included two unidentified *Haemoproteus* sequences from a Red-headed woodpecker (*Melanerpes erythrocephalus*) (AF465590) and a tropical member of Piciformes (*Indicator maculatus*) (EU810718). A partial cyt *b* sequence from *Leucocytozoon squamatus* (DQ4514320) was used as an outgroup. Our ML phylogenetic construction used a GTR + I+Γ model based on an AICc analysis of models and 1000 bootstrap replicates.

## 3 Results

### 3.1 White-headed woodpecker fatality

The juvenile White-headed woodpecker was radio-tagged at its nest site on 26 June 2015 before released beingback into the nest to fledge naturally. The estimated fledge date was 30 June 2015. We tracked the bird on 12 different days in July before tracking its radio signal led us to locate it dead on the ground, without obvious signs of trauma, on 21 July 2015. It was not observed begging for food from parents in the characteristically loud and vocal manner of recently fledged White-headed woodpeckers. Prior to its death, this juvenile was relatively sedentary compared to other radio-tagged juveniles, the entire area it ranged over during the 22 days before its death was 6.4 ha (ha). This is the smallest range observed for 54 juveniles tracked during their dependence period (mean range = 252.8 ha, SD ± 94.9 ha).

**Fig. 1:**
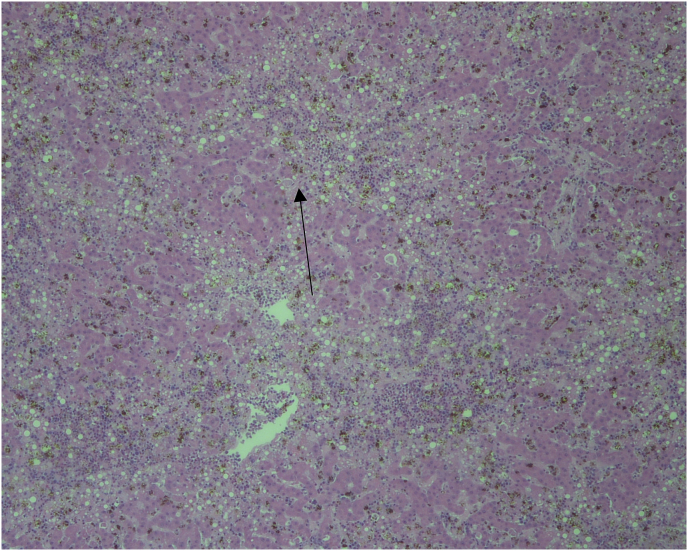
Histological section of liver tissue of naturally infected White-headed woodpecker (*Dryobates albolarvatus*). Note clumps of numerous hemozoin granules (arrows), which are remnants of hemozoin developing in gametocytes of *Haemoproteus* parasites. Magnification x10.

There were several macroscopic findings that pointed to an infectious disease cause of death. The breast muscle was paler than normal with multifocal white streaks throughout and the distal half of both lungs were congested. Lymphatic observations showed the spleen was three to four times the normal size. The liver had green discoloration and was severely enlarged, extending beyond the last rib. Histology revealed acute multifocal necrosis, disseminated moderate to severe microgranulomatosis, lymphohistiocytosis in the liver and spleen as well as in kidney and skeletal muscle. Iron sequestration due to the presence of numerous pigment granules (hemozoin) was seen in the liver (Fig. 1).

**Fig. 2:**
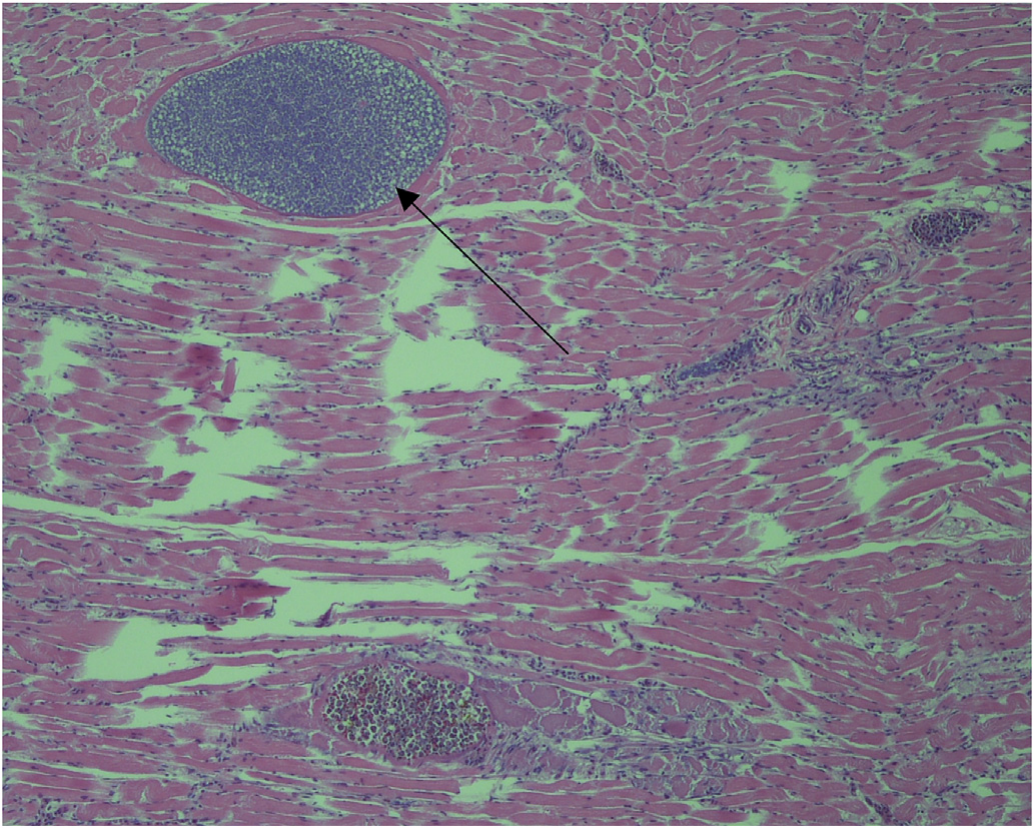
Skeletal muscle tissue showing megalomeronts of *Haemoproteus velans*. Note numerous developing cytomeres (arrowhead) and capsular-like wall around the parasite (arrow). Magnification x20.

Histological examination revealed that megalomeronts were present within the cytoplasm of capillary endothelial cells, hepatocytes, and myocytes, or free in the tissues (Fig. 2). Electron microscope observations in other organs include numerous large, basophilic granular bodies within the wall of the blood vessels, pulmonary parabronchi, in the hepatic sinusoids, cytoplasm of macrophages, and between the myofibers of the skeletal muscle. Electron microscopy also showed protozoal young gametocytes within the cytoplasm of erythrocytes. Gametocytes had a double cell membrane and were slightly eccentric.

### 3.2 Northern Flicker *Haemoproteus velans* identification

Sampling from 139 live birds in 2016 and 2017 revealed three adult Northern Flickers with *Haemoproteus* infections. All three samples had an identical genetic sequences and infections were confirmed with microscopy. The genetic sequence was two base pairs different or 0.417% divergent from the sequence of the White-headed woodpecker. When morphologically comparing gametocytes in blood smears from individuals from this study and from the Downy woodpecker (EU24552), all samples were identified as *H. velans*. There were no blood smears available from the White-headed woodpecker. Given the genetic similarity *Haemoproteus* parasites in the Northern Flicker, White-headed woodpecker, and Downy woodpecker samples and also the morphological similarity of gametocytes from the Northern Flicker and Downy woodpecker, it is probable that all records of haemoproteids in these birds were *H. velans*.

**Fig. 3:**
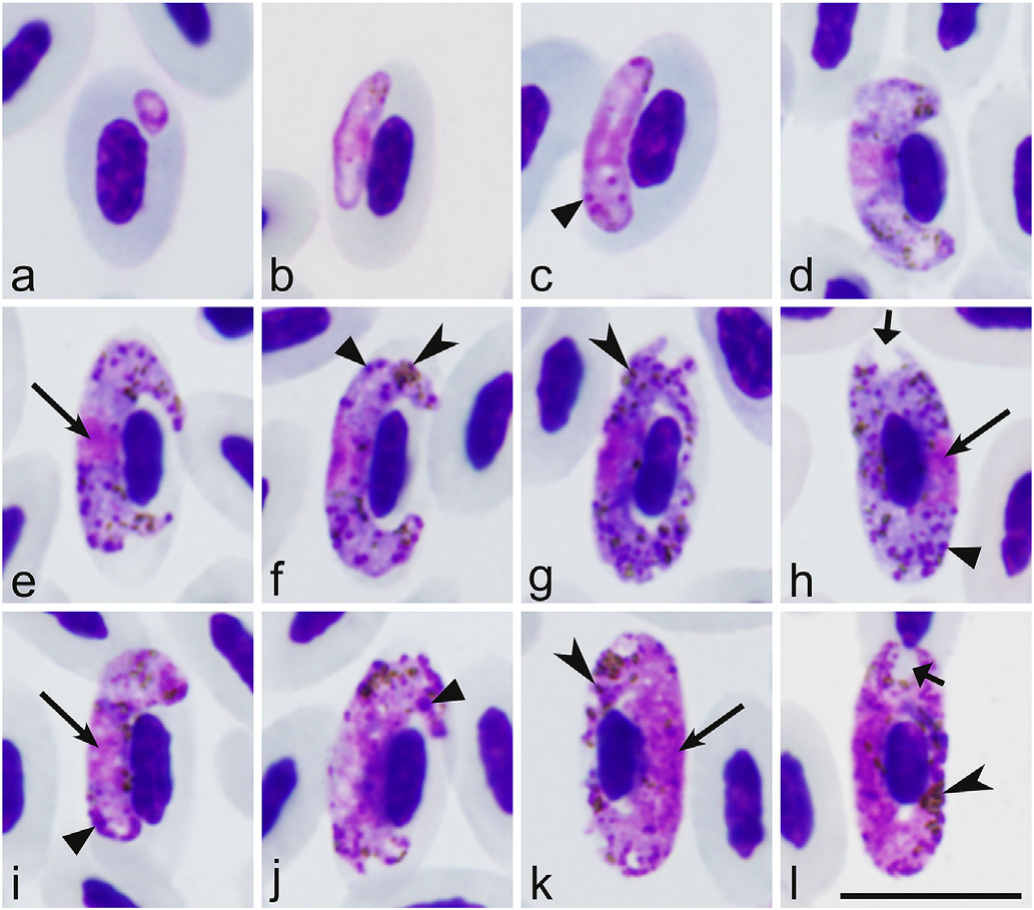
*Haemoproteus velans* (lineage NOFL1) from the blood of the Northern Flicker (*Colaptes auratus*): a, b - young gametocytes; c-h - macrogametocytes; i-l - microgametocytes. Long simple arrows – nuclei of parasites. Simple arrowhead – pigment granules. Triangle arrowheads – volutin granules. Short simple wide arrow – unfilled space in poles of erythrocytes. Giemsa-stained thin blood films. Scale bar = 10 μm.

Only one morphotype was present in all three of the positive Northern Flicker slides examined (Fig. 3). The original description of *H. velans* is fragmentary, and morphometric data are incomplete (Coatney and Roudabush, 1937; Greiner et al., 1977; Valkiūnas, 2005). Here, we provide additional information on the morphology of this parasite (Fig. 3) and morphometrics of its fully-grown gametocytes and their host cells (Table 2) from the type vertebrate host, the Northern Flicker. It should be noted that blood stages were identical in the main morphological features to those described by Coatney and Roudabush (1937) and Valkiūnas (2005), their description is not repeated here. However, several additional features in gametocyte morphology were reported. First, there were more amoeboid gametocyte cells in some individual avian hosts than in previous descriptions (see Fig. 3g, k). Second, volutin was readily visible, but present markedly unequally in individual birds. Third, the number of pigment granules is greater in our material than in the original description. Due to marked volutinization of parasite cytoplasm, it is often difficult to calculate the number of pigment granules in gametocytes of this parasite (Valkiūnas, 2005). Gametocytes in the type material of *H. velans* are overfilled with prominent volutin, and pigment granules are poorly visible due to marked fading of hemozoin in these old preparations (Dimitrov et al., 2014). We calculated pigment granules in mature gametocytes with a relatively small amount of volutin, and these data show that mature gametocytes contain on average approximately 35 and 28 pigment granules in macro-and microgametocytes, respectively (Table 2). This is greater than was reported in the original description, in which 18 and 21 pigment granules were reported on average in macro- and microgametocytes, respectively (Coatney and Roudabush, 1937; Valkiūnas, 2005).

**Table 2:** Morphometry of host cells and mature gametocytes of *Haemoproteus velans* from the blood of the Northern Flicker *Colaptes auratus.*

Feature	Measurements (μm)^a^
Uninfected erythrocyte	
Length	12.6–14.6 (13.5 ± 0.6)
Width	6.3–7.5 (6.7 ± 0.3)
Area	65.8–77.9 (71.7 ± 3.3)
Uninfected erythrocyte nucleus	
Length	5.3–6.6 (6.1 ± 0.3)
Width	1.9–2.6 (2.2 ± 0.2)
Area	9.0–13.5 (11.5 ± 1.3)
Macrogametocyte	
Infected erythrocyte	
Length	14.5–17.8 (15.9 ± 0.8)
Width	5.9–7.2 (6.6 ± 0.4)
Area	78.7–97.4 (85.0 ± 5.4)
Infected erythrocyte nucleus	
Length	4.9–6.7 (5.9 ± 0.4)
Width	1.9–2.4 (2.2 ± 0.2)
Area	8.1–12.9 (11.2 ± 1.1)
Gametocyte	
Length	24.1–27.5 (25.9 ± 1.0)
Width	1.9–3.7 (2.6 ± 0.4)
Area	57.7–77.6 (65.5 ± 5.4)
Gametocyte nucleus	
Length	2.8–4.9 (4.0 ± 0.6)
Width	1.7–3.5 (2.3 ± 0.5)
Area	5.9–11.6 (8.1 ± 1.4)
Pigment granules	27.0–42.0 (34.9 ± 4.3)
NDR^b^	0.4–0.9 (0.8 ± 0.1)
Microgametocyte	
Infected erythrocyte	
Length	14.4–17.1 (15.4 ± 0.7)
Width	5.8–7.3 (6.6 ± 0.3)
Area	72.6–95.8 (83.8 ± 5.2)
Infected erythrocyte nucleus	
Length	4.9–6.6 (5.7 ± 0.4)
Width	1.7–2.6 (2.3 ± 0.2)
Area	9.3–12.7 (11.0 ± 1.0)
Gametocyte	
Length	22.3–28.8 (25.0 ± 1.7)
Width	2.3–3.1 (2.8 ± 0.2)
Area	50.7–72.0 (62.1 ± 5.4)
Gametocyte nucleus c	
Length	6.7–9.5 (8.3 ± 1.0)
Width	2.3–3.1 (2.6 ± 0.3)
Area	14.5–24.9 (19.6 ± 3.8)
Pigment granules	19.0–36.0 (27.5 ± 4.8)
NDR	0.6–0.8 (0.7 ± 0.1)

a Measurements (n = 21) are given in micrometers. Minimum and maximum values are provided, followed in parentheses by the arithmetic mean and standard deviation.

b NDR = nucleus displacement ration according to Bennett and Campbell (1972).

c Nuclei of microgametocytes are markedly diffuse and difficult to measure; outline of the nuclei was well seen and measured in 9 microgametocytes.

**Fig. 4:**
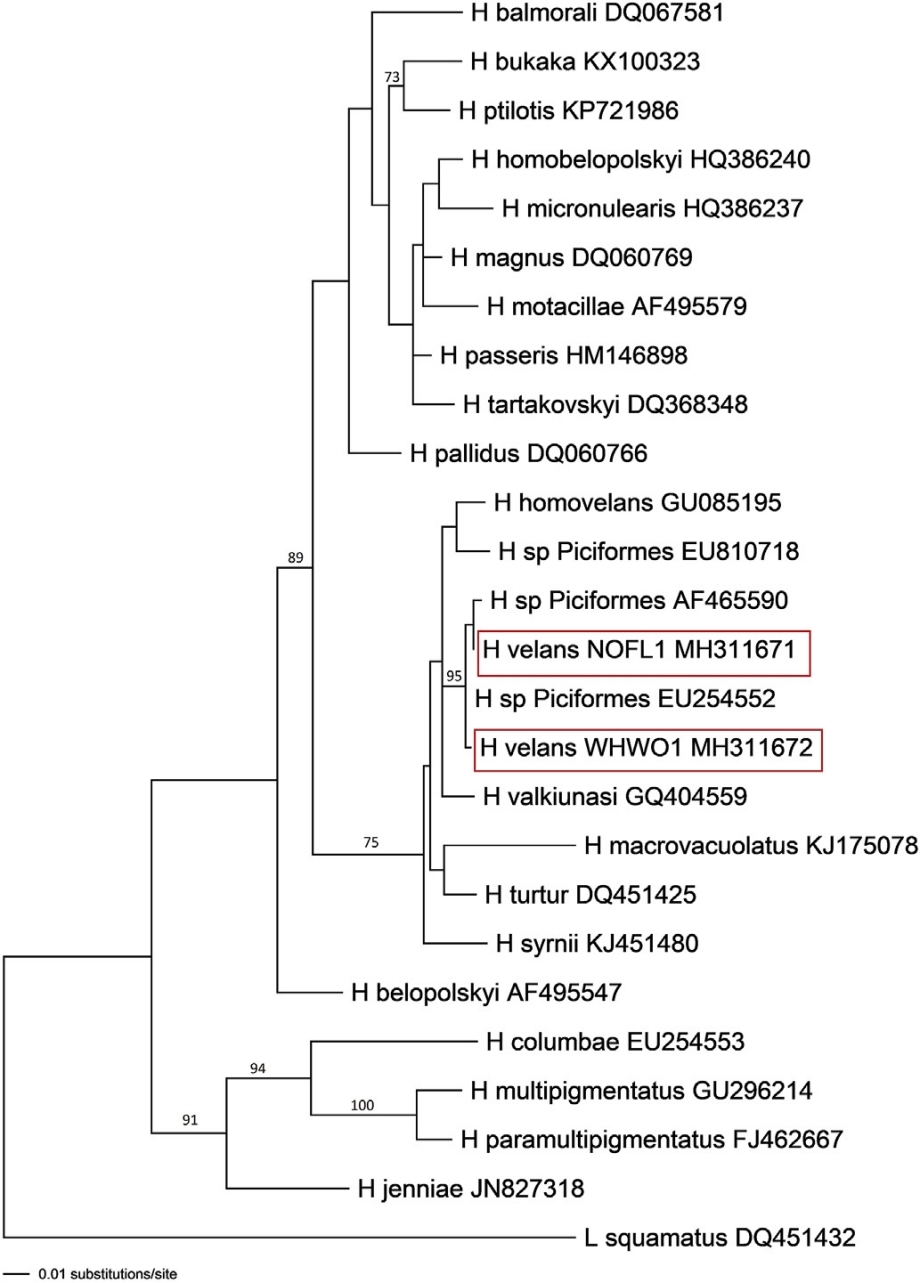
Consensus tree displaying *Haemoproteus velans* phylogenetic relationships as predicted by Maximum-likelihood inference, using GTR + I+Γ substitution model in PAUP* v.4.0a.b161. Maximum-likelihood bootstrap values > 70 are indicated. Lineages detected in the current study are indicated by red boxes. (For interpretation of the references to color in this figure legend, the reader is referred to the Web version of this article.)

Maximum-likelihood predictions placed *H. velans* into a clade with *Haemoproteus* species parasitizing non-passeriform birds belonging to Strigiformes, Ciconiiformes, Columbiformes, and European members of Piciformes with 96% bootstrap support (Fig. 4). However, the relationships between these species did not have significant bootstrap support. The NOFL1 (Northern Flicker) and WHWO1 (White-headed woodpecker) lineages of *H. velans* formed a clade with 100% bootstrap support with the likely misidentified sequence from the Downy woodpecker (EU254552*, H. picae*) and the unidentified *Haemoproteus* sequence from the Red-headed woodpecker (AF465590).

## 4 Discussion

*Haemoproteus velans* infection can result in fatalities in woodpeckers, but the frequency of mortality remains unclear. Haemosporidian infections of any kind have not been described as a source of mortality in White-headed woodpeckers in past publications. Due to their sensitive status and concerns about impacting nest success, no blood samples have previously been taken from this species. The three positive adult Northern Flickers detected during sampling in 2016 and 2017 coupled with a likely historical prevalence of 24.9% (Table 1), indicates that *H. velans* can persist chronically in adult woodpecker populations. Work by Schrader et al. (2003) found a prevalence of 25% of *H. velans* in Red-bellied woodpeckers (*Melanerpes carolinus*) and showed a seasonal variation in the proportion of individuals infected. Results from monitoring infected adults suggests that infection with *H. velans* may not directly affect adult host survival but can negatively affect host condition (Schrader et al., 2003). Experimental observations indicate that juvenile chaffinches (*Fringilla coelebs)* are less mobile during peak parasitemia in comparison to non-infected individuals and are thus more attractive to predators (Valkiūnas, 2005). Additionally, *Haemoproteus* spp. infected Blackcap (*Sylvia atricalilla*) nestlings lost weight during peak parasitemia (Valkiūnas et al., 2006). Nestlings sampled in this study were 20–24 days old may not have had infections detected due to the infection being in the prepatent period and not detectable yet in the blood. These data and the observations in this study indicate that *Haemoproteus* infections may be more virulent than formerly believed (Bennett et al., 1993b). However, more sensitive epidemiology approaches than microscopic examination or PCR-based blood testing are needed to recognize true host-parasite relationships in wildlife. This study shows how the application of radio-tagging can be a useful methodology in such research.

Necropsy results of the White-headed woodpecker attributed the cause of death to decreased liver and spleen function and a high number of infected erythrocytes which lead to hypoxia. The streaking patterns seen in the muscle tissue are similar to findings by Cardona et al. (2002) in captive Bobwhite Quail (*Colinius virginsius)* infected with *H. lophortyx* and in experimentally infected turkeys (*Meleagris gallapavo*) infected with *H. mansoni* (syn. *H. meleagridis*) (Atkinson et al., 2008)*.* The prepatent period of tissue stage development of the majority of *Haemoproteus* species studied varies from 11 days to three weeks (Valkiūnas, 2005; Atkinson et al., 2008). Since the juvenile was found dead 22 days after fledgling, it was probably infected after it left the nest. It was not exhibiting normal juvenile behavior. Coupled with the significantly lower home range (mean range = 252.8 ha, SD ± 94.9 ha) than other juveniles, these observations suggest this infection altered its behavior prior to death.

Other eukaryotic parasites were not reported in the dead juvenile. It is conceivable that *H. velans* may only cause mortality when a bird is already subject to other health impacts. It is possible the White-headed woodpecker juvenile had a viral or bacterial infection not detected during necropsy. Testing PCR material for additional pathogens was beyond the scope of this study. Environmental conditions could also have affected the juvenile's overall health. The mortality occurred on 20 July 2015 and followed the warmest June and January–June period for the eastern Washington Cascades ever reported, as determined by climatological data from 1895 to 2015 (NOAA, 2015). An increase in temperature may have resulted in decreased or shifted foraging opportunities for adults, leading to decreased overall health of their chicks (Walther et al., 2002). These factors might have contributed to the death of the individual. It would be important to study how the accumulation of several health factors simultaneously affect overall immune health and contribute to the pathogenicity of haemosporidians.

This study provides the first molecular characterization of *H. velans*, providing opportunities for better disease diagnostics in the wild. The genetic similarity between the White-headed woodpecker and Norther Flicker sequences (0.417%) indicates they are potentially different lineages from the same species of parasite (Martinsen et al., 2006; Hellgren et al., 2007). Morphological characterization (Fig. 3, Table 2) is valuable for easier identification of this infection during microscopic examination of blood films. Morphological studies combined with molecular characterization of parasite species in different host individuals from a variety of locales can help better understand intraspecies variation. The higher frequency of amoeboid gametocyte cells than in previous descriptions (see Fig. 3g, k) shows that this character should be used carefully during species identification as the frequency depends on the stage of gametocytemia. The amount of volutin in gametocytes of *H. velans* also likely depends on the stage of gametocyte development and probably on the individual host thus, this character is not as dependable in this parasite species identification as previously though (Coatney and Roudabush, 1937; Valkiūnas, 2005). This is important for distinguishing abortive haemosporidian infections, which might cause severe diseases in birds (Valkiūnas and Iezhova, 2017).

The phylogenetic relationship of *H. velans* to other *Haemoproteus* species has low bootstrap support in our maximum-likelihood analysis. This is likely due the lack of sufficient informative information associated with the 480 bp cyt *b* fragment used in our analysis. Despite the low bootstrap support, the relationships between *Haemoproteus* species in this study are generally the same as those found by Pacheco et al. (2018) in their analysis of the whole mitochondrial genome. Though longer sequences may be necessary to understand complex phylogenetic relationships, the partial cyt *b* sequence used appears to be sufficient for ecological investigations (Pacheco et al., 2018). There is extensive data available on this fragment and it is the current standard in avian haemosporidian studies (Bensch et al., 2009; Outlaw and Ricklefs, 2014).

Our data led to a likely species identification of the unidentified *Haemoproteus* sequence from the Red-headed woodpecker (AF465590) as *H. velans* due to its clustering in a clade with lineages from this study as well as a likely misidentified parasite from a Downy woodpecker (EU254552). This indicates that they likely all belong to *H. velans* and that *H. velans* is distributed across North America. An analysis by Valkiūnas et al. (2008a, b) showed that most haemosporidian lineages deposited in GenBank only have a genus level identification. These are not useful when investigating the life histories and phylogenetic relationships (Valkiūnas et al., 2008a), particularly with the broad distribution of abortive haemosporidian infections (Moens et al., 2016; Valkiūnas and Iezhova, 2017). There are also a number of incorrectly identified haemosporidian parasite DNA sequences in GenBank (Valkiūnas et al., 2008a). The genetic sequence from the White-headed woodpecker fatality matched the genetic sequence of parasites morphologically identified as *H. picae* in a Downy woodpecker (EU254552) (Martinsen et al., 2008). Dimitrov et al. (2014) suspected it to be incorrectly identified since *H. picae* previously only infected species in the passerine family Corvidae (Valkiūnas, 2005), and it is unlikely it can complete its life cycle and produce gametocytes in birds of Piciformes, as suggested by Martinsen et al. (2008). Mature *H. velans* gametocytes (Fig. 3h, l) completely surround the nucleus of erythrocytes occupying all available cytoplasmic space (Greiner et al., 1977; Valkiūnas, 2005) and this is not found in *H. picae* (Valkiūnas, 2005). Molecular data (Fig. 4) supported the conclusion the cyt *b* sequence EU254552 likely belongs to *H. velans*. This misidentification created confusion on the part of the authors of this paper when trying to identify the parasites from this study.

Radio-tagging provides valuable information about the fate of infected vertebrates. It is worth a broader use in parasitology and ecology research where it is difficult to access sick individuals. Monitoring radio-tagged individuals can show how haemosporidians and other parasites affect activity levels and behavior in real time. Importantly, fresh tissue from dead birds can be collected using this methodology which is essential for histopathology research. This study is the first to document what happens to a sick radio-tagged bird and shows that radio-tagging can be a useful methodology in future research on pathogenicity of haemosporidians.

As climate and land use change continues to shift suitable habitat ranges for many bird species (Langham et al., 2015), the risk and severity of infectious diseases are projected to increase (Patz et al., 2000; Garamszegi, 2011). Future models of temperature and precipitation patterns reveal potential dramatic changes in coming years (Langham et al., 2015), a particular concern for species that are already rare, declining, or otherwise of conservation concern. The population health of indicator species such as woodpeckers, which have a disproportionate effect on ecosystems they inhabit (Virkkala, 2006), becomes an important measure of how the rest of the ecosystem is reacting to these changes (Lindenmayer et al., 2000). Haemoproteosis remains a neglected avian parasitosis. Due to pathology associated with tissue stages, non-adapted avian species already experiencing population declines may be at risk (Valkiūnas and Iezhova, 2017). Identifying the effects of haemosporidian infections on woodpecker population health could contribute to stronger management of an important keystone guild.

## Declaration of interest

None.
